# Bringing state-of-the-art diagnostics to vulnerable populations: The use of a mobile screening unit in active case finding for tuberculosis in Palawan, the Philippines

**DOI:** 10.1371/journal.pone.0171310

**Published:** 2017-02-02

**Authors:** Fukushi Morishita, Anna Marie Celina Gonzales Garfin, Woojin Lew, Kyung Hyun Oh, Rajendra-Prasad Yadav, Janeth Cuencaho Reston, Lenie Lucio Infante, Maria Rebethia Crueldad Acala, Dean Lim Palanca, Hee Jin Kim, Nobuyuki Nishikiori

**Affiliations:** 1 World Health Organization Regional Office for the Western Pacific, Manila, Philippines; 2 National TB Control Programme, Department of Health, Manila, Philippines; 3 World Health Organization Representative Office in Mongolia, Ulaanbaatar, Mongolia; 4 Korean Institute of Tuberculosis, Cheongju, Republic of Korea; 5 World Health Organization Representative Office in the Philippines, Manila, Philippines; 6 Provincial Health Office of Palawan, Puerto Princesa, Philippines; 7 City Health Office of Puerto Princesa City, Puerto Princesa, Philippines; McGill University, CANADA

## Abstract

**Background:**

Globally, case detection of tuberculosis (TB) has stabilized in recent years. Active case finding (ACF) has regained an increased attention as a complementary strategy to fill the case detection gap. In the Philippines, the DetecTB project implemented an innovative ACF strategy that offered a one-stop diagnostic service with a mobile unit equipped with enhanced diagnostic tools including chest X-ray (CXR) and Xpert^®^MTB/RIF (Xpert). The project targeted the rural poor, the urban poor, prison inmates, indigenous population and high school students.

**Methods:**

This is a retrospective review of TB screening data from 25,103 individuals. A descriptive analysis was carried out to compare screening and treatment outcomes across target populations. Univariate and multivariate analyses were performed to identify predictors of TB for each population. The composition of bacteriologically-confirmed cases by smear and symptom status was further investigated.

**Results:**

The highest yield with lowest number needed to screen (NNS) was found in prison (6.2%, NNS: 16), followed by indigenous population (2.9%, NNS: 34), the rural poor (2.2%, NNS: 45), the urban poor (2.1%, NNS: 48), and high school (0.2%, NNS: 495). The treatment success rate for all populations was high with 89.5% in rifampicin-susceptible patients and 83.3% in rifampicin-resistant patients. A relatively higher loss to follow-up rate was observed in indigenous population (7.5%) and the rural poor (6.4%). Only cough more than two weeks showed a significant association with TB diagnosis in all target populations (Adjusted Odds Ratio ranging from 1.71 to 6.73) while other symptoms and demographic factors varied in their strength of association. The urban poor had the highest proportion of smear-positive patients with cough more than two weeks (72.0%). The proportion of smear-negative (Xpert-positive) patients without cough more than two weeks was the highest in indigenous population (39.3%), followed by prison inmates (27.7%), and the rural poor (22.8%).

**Conclusions:**

The innovative ACF strategy using mobile unit yielded a substantial number of TB patients and achieved successful treatment outcomes. TB screening in prison, indigenous population, and urban and rural poor communities was found to be effective. The combined use of CXR and Xpert largely contributed to increased case detection.

## Introduction

Globally, case detection of TB has stabilized in recent years, and 4.3 million cases are estimated to be either not diagnosed or not notified [1]. Limitations of the DOTS strategy which has focused on passive case finding (PCF) by smear microscopy among symptomatic individuals have been increasingly recognized [2–4]. In response to this, targeted active case finding (ACF) has regained an increased attention as a complementary strategy to fill the case detection gap [5,6]. For the last one and half decades, large donor funds have been mobilized worldwide and a number of ACF projects have been implemented in high and intermediate burden countries to stimulate and gather global evidence [7]. The World Health Organization (WHO) facilitated a review of available evidence and provided principles and recommendations on systematic screening for active TB [8]. In principle, systematic screening including ACF should target specific groups that are at high risk of TB or have poor access to TB services, or both [9]. According to the WHO guidelines, it is strongly recommended for people living with human immunodeficiency virus (HIV), close contacts of people with TB, and people exposed to silica, and conditionally recommended for other groups such as prisoners, migrants, people with diabetes mellitus, and urban slums dwellers [8].

Monitoring and evaluation (M&E) is an integral part of any screening programme [9]. A set of basic M&E indicators has been proposed, which includes acceptance rate, retention rate for each screening step, number needed to screen (NNS) and number needed to test (NNT) to detect one case, treatment initiation rate, and treatment success rate (TSR) [9]. In particular, NNS, defined as the number of persons screened divided by the number of persons diagnosed with TB (roughly the inverse of the prevalence), is increasingly used to quantify the effectiveness of screening programmes [9]. In a systematic review on NNS in TB screening, NNS of 100 or lower, in high-incidence settings, was found in several groups such as people living with HIV (PLHIV) including voluntary counselling and testing attendees (NNS 10–37), household contacts (NNS 17–25), drug users (NNS = 20), persons with diabetes (NNS = 35), miners (NNS = 36), and certain community-wide screening in high-incidence countries (NNS = 100) [10]. However the review concluded that NNS is highly variable depending on the different screening approaches and population evaluated [10]. As such, NNS can be more useful when comparing it across different groups in a setting with a fixed screening algorithm, which provides a measure of relative cost-effectiveness [9]. In Nepal, an intensified case finding project screened multiple risk groups for TB using mobile vans that equipped with microscopy and Xpert^®^MTB/RIF (Xpert) with or without symptom screening. The project had high yield and low NNS in PLHIV (NNS: 17) and household contacts (NNS: 29), followed by urban slum dwellers (NNS: 197) and prisoners (NNS: 261) [11]. Given the increasingly diversified approaches and targets in ACF, individual project assessment is critical to add to evidence-based policy guidance.

When ACF is pursued, it is essential to ensure local health systems have adequate capacity to provide quality TB treatment and care and to respond to the anticipated rise in case detection [8,9]. TSR is an important indicator to monitor and evaluate this capacity. Studies have increasingly reported a high TSR in ACF with more than 85%, being at a similar or higher level as compared to baseline or routine PCF [12–14]. However, careful assessment is needed since several studies showed that ACF led to more unfavourable treatment outcomes due to a high proportion of less symptomatic patients who may be less motivated to complete their treatment [15,16].

The Philippines is one of 30 countries with high burdens of TB, with the estimated incidence rate of 322 per 100,000 population in 2015 [1]. The country also has one of the highest burden of multi-drug resistant (MDR) TB [1]. Case notification has steadily increased since 2008 after eight years of stagnation, however the gap still appears to be substantial [17]. With the increased initiative of the National TB Control Programme (NTP), significant progress has been made in TB control for the past decades. Nationwide DOTS coverage was achieved in 2003 [18]. Programmatic efforts have been undertaken for establishing partnerships with public and private health providers, facilitating nationwide expansion of TB diagnosis in children, and scaling-up Programmatic Management of Drug-resistant TB (PMDT) [18]. More recently, the NTP has been active in bringing national and international partners together to set up interventions including ACF to address the needs of vulnerable populations. However, evidence to suggest the effectiveness of ACF in the Philippines is insufficient.

Since 2012, the DetecTB project has been implemented in the province of Palawan, aiming to increase TB case detection among vulnerable populations. The project implemented an innovative ACF strategy that offered a one-stop diagnostic service with the use of a mobile unit and enhanced diagnostic tools including chest X-ray (CXR) and Xpert. The ACF targeted five vulnerable populations including; (1) residents in rural poor communities, (2) residents in urban poor communities, (3) prison inmates, (4) indigenous population, and (5) high school students. This study primarily aimed to describe yield of TB diagnosis and treatment outcomes by different target populations. Besides, we further examined predictors of TB and the contribution of enhanced diagnostic tools to overall case detection.

## Methods

### Programmatic information

The DetecTB project has been implemented in Palawan, with the first phase from December 2012 to March 2014 and the second phase from April 2014 to the present. The project was funded by the Korea Foundation for International Healthcare (KOFIH) and implemented in collaboration with the Department of Health of the Philippines, Korean Institute of Tuberculosis (KIT), WHO, and the local government units of Palawan Province and Puerto Princesa City.

Of 23 municipalities and one city in Palawan Province, seven municipalities were selected based on health access and the level of TB case detection, and classified as rural poor communities. Additionally, of 66 barangays (the smallest administrative division) in Puerto Princesa City, three barangays were selected based on the income levels in the official statistics, and classified as urban poor communities. The project also targeted two major indigenous populations (the Palawano peoples in the selected seven municipalities and the Batak tribes in additional three barangays in Puerto Princesa City), inmates from six prison facilities (two jails and the national prison that has four sub-colonies located in Puerto Princesa City), and students from eight high schools.

The project delivered the TB screening and diagnostic services to all target populations through a mobile unit. The Provincial Health Office of Palawan and City Health Office of Puerto Princesa City each formed a mobile team which was composed of two physicians, one nurse, one medical technologist and one radiologic technologist. They were trained on the use of mobile unit for two weeks in the KIT prior to the implementation of the project.

The mobile team utilized a service bus to travel from one project site to another. The mobile unit was equipped with all diagnostic equipment including a digital CXR machine, Light Emitting Diode Fluorescence Microscope (LED-FM), and Xpert machine for molecular diagnosis. The mobile unit also had a slide warmer, biosafety cabinet, and refrigerator to temporarily store sputum specimens. The mobile unit had a capacity to screen over 250 individuals per day at a maximum while it screened around 50–100 individuals per day on average.

ACF activities were coordinated in close collaboration with a point person in each target site (i.e. municipal health officers, barangay health workers, prison/school officials, etc.). They undertook the field preparations including information dissemination and advocacy meetings prior to the pre-scheduled screening sessions. Local health workers were also pre-oriented to the case finding process, and assisted the mobile teams in organizing participants, obtaining consents, conducting interviews, smearing slides, and providing health education.

In the targeted prisons and high schools, all eligible persons were screened by the project. On the other hand, in rural and urban poor communities and indigenous population, only those who voluntarily visited the mobile unit were screened.

During the screening session, all participants aged 15 or above were interviewed for TB symptoms and the known risk factors for TB such as history of TB exposure and previous TB treatment, using a standardized screening form. All interviewed participants then underwent CXR examination. CXR results were read by the physician in the mobile team who were trained on CXR reading by the KIT. The results were classified into five categories; “normal”, suggestive of “active TB” and “inactive TB”, “TB activity undetermined” and “other diseases”. People with suspected TB were determined by clinical judgement of physicians, based primarily on abnormal CXR results (such as “active TB” and “TB activity undetermined”) and additionally on the presence of TB symptoms. They were requested to provide two spot sputum specimens for diagnostic tests. At least one hour interval was given between the first and second specimen collection. Both specimens were examined by sputum smear microscopy using LED-FM, and one of them was tested by Xpert by the trained medical technologist. The test results were released on the same day. Those who had a positive result by smear microscopy and/or an Xpert result with “Mycobacterium Tuberculosis (MTB) detected” were diagnosed as bacteriologically-confirmed TB. Those who had negative sputum microscopy and Xpert results but had strong clinical evidence for active TB, based on presenting signs and symptoms and risk factors, were also treated for TB according to the physician’s best clinical judgment. The project focused on diagnosing only pulmonary TB, but not extra-pulmonary TB.

Both bacteriologically-confirmed and clinically-diagnosed TB patients were referred to local treatment centres for appropriate patient management in line with the national guidelines. CXR results of patients found to have rifampicin resistance (RR) TB were sent a radiologist for reconfirmation, and then they were referred to the PMDT facility for treatment.

Treatment outcomes were reported in line with the national TB guidelines [17] and the latest World Health Organisation's definitions [19]. Treatment success is defined as the sum of “cured” and “completed”.

### Study design, analysis and ethical considerations

This is a retrospective review of the screening data from the DetecTB project that covers the period from December 2012 to August 2015.

All raw data were obtained from a paper-based screening form used in the screening session, and then entered into an Epi Info 7 SQL database (CDC Atlanta Georgia, USA) with predefined coding and error-checking formulas. Data processing and statistical analyses were performed using R3.3.0 (CRAN: Comprehensive R Archive Network at https://cran.r-project.org/).

A descriptive analysis was carried out by computing distribution and frequency of screening outcomes and demographic characteristics of participants by different target population. A univariate analysis was performed to identify predictors of TB for detected patients in the rural poor, the urban poor, prison inmates, and indigenous population. Using the variables deemed significant in the univariate analysis and sex and age category as priori variables, a multivariate logistic regression analysis was performed to confirm the observed association. An odds ratio (OR) and adjusted odds ratio (AOR) were calculated to examine the strength of the association, along with 95% Confidence Interval (CI). Statistical significance was set at p <0.05. To see the contribution of CXR and Xpert to overall case detection, we further examined the composition of bacteriologically-confirmed TB cases by smear and symptom status for each target population. Treatment outcomes were then computed, stratified by rifampicin resistant status, excluding patients with ongoing treatment.

As stipulated by the project protocol, a written informed consent was obtained from all screened subjects. If participants were minor, a written informed consent was obtained from their guardians. Patient identifiable information was removed from the dataset before analysis. As this study was part of the routine assessment of the ongoing project by using existing records, ethical clearance was not required according to local regulations.

## Results

### Characteristics of participants (Table 1)

**Table 1:** Characteristics of participants by target population.

Characteristics		Rural poor		Urban poor		Prison inmates		Indigenous population		High school students		Total	
		N = 12907	(%)	N = 1625	(%)	N = 6133	(%)	N = 2145	(%)	N = 2293	(%)	N = 25103	(%)
Sex	Female	8055	(62.4%)	905	(55.7%)	66	(1.1%)	1376	(64.1%)	1337	(58.3%)	11739	(46.8%)
	Male	4852	(37.6%)	720	(44.3%)	6067	(98.9%)	769	(35.9%)	956	(41.7%)	13364	(53.2%)
Age	Median	46.0	(IQR:33–59)	40.0	(IQR:26–52)	41.0	(IQR:33–49)	44.0	(IQR:32–58)	16.0	(IQR:15–16)	42.0	(IQR:28–54)
	Mean	46.5	(SD:17.3)	40.5	(SD:16.7)	41.3	(SD:11.6)	45.2	(SD:17.1)	15.8	(SD:1)	41.9	(SD:17.4)
Age category	15–24	1541	(11.9%)	346	(21.3%)	439	(7.2%)	270	(12.6%)	2293	(100.0%)	4889	(19.5%)
	25–34	1991	(15.4%)	298	(18.3%)	1443	(23.5%)	376	(17.5%)	0	(0.0%)	4108	(16.4%)
	35–44	2465	(19.1%)	341	(21.0%)	1878	(30.6%)	447	(20.8%)	0	(0.0%)	5131	(20.4%)
	45–54	2491	(19.3%)	282	(17.4%)	1535	(25.0%)	410	(19.1%)	0	(0.0%)	4718	(18.8%)
	55–64	2214	(17.2%)	205	(12.6%)	699	(11.4%)	325	(15.2%)	0	(0.0%)	3443	(13.7%)
	≥65	2205	(17.1%)	153	(9.4%)	139	(2.3%)	317	(14.8%)	0	(0.0%)	2814	(11.2%)
History of TB exposure*	No	10007	(77.8%)	1559	(96.1%)	4647	(75.9%)	1714	(80.3%)	2212	(96.6%)	20139	(80.5%)
	Yes	2853	(22.2%)	63	(3.9%)	1472	(24.1%)	421	(19.7%)	79	(3.4%)	4888	(19.5%)
History of previous TB treatment*	No	11624	(90.6%)	1498	(92.6%)	5189	(84.8%)	1930	(91.1%)	2266	(98.9%)	22507	(90.1%)
	Yes	1213	(9.4%)	120	(7.4%)	931	(15.2%)	189	(8.9%)	25	(1.1%)	2478	(9.9%)
Smoking status*	No	7595	(71.0%)	770	(64.1%)	852	(17.6%)	991	(67.4%)	1712	(86.8%)	11920	(59.1%)
	Yes	3103	(29.0%)	431	(35.9%)	3980	(82.4%)	479	(32.6%)	260	(13.2%)	8253	(40.9%)
Location	Palawan	12907	(100.0%)	0	(0.0%)	0	(0.0%)	1934	(90.2%)	326	(14.2%)	15167	(60.4%)
	Puerto Princesa	0	(0.0%)	1625	(100.0%)	6133	(100.0%)	211	(9.8%)	1967	(85.8%)	9936	(39.6%)

* Cases with unknown or missing information were excluded from the calculation.

IQR: Interquartile Range, SD: Standard Deviation

A total of 25103 individuals were screened by the project during the study period. Of them, 12907 were screened from rural poor communities; 1625 from urban poor communities; 6133 from prisons, 2145 from indigenous population; and 2293 from high schools.

The characteristics of the participants were highly heterogeneous across different target populations. For example, males were predominant among prison inmates (98.9% vs 1.1%) whereas more females than males were screened among indigenous population (64.1% vs 35.9%). Participants from high schools were young with the median age of 16 while other groups had their median age of 40 or over. Approximately 20% of participants had a history of TB exposure (close contact within the past two years) in the rural poor, prison inmates and indigenous population whereas only 3–4% were previously exposed to TB in the urban poor and high school students. The proportion of participants with a history of previous TB treatment was the highest in prison inmates (15.2%), which was followed by the rural poor (9.4%), indigenous population (8.9%), and the urban poor (7.4%). The prevalence of smoking was the highest in prison inmates (82.4%), followed by the urban poor (35.9%), indigenous population (32.6%) and the rural poor (29.0%).

### Outcomes of screening tests (Table 2)

**Table 2:** Screening and diagnostic outcomes by target population.

Screening and diagnostic outcomes		Rural poor		Urban poor		Prison inmates		Indigenous population		High school students		Total	
		N = 12907	(%)	N = 1625	(%)	N = 6133	(%)	N = 2145	(%)	N = 2293	(%)	N = 25103	(%)
Any TB symptoms	None	2582	(20.0%)	906	(55.8%)	2184	(35.6%)	266	(12.4%)	1688	(73.6%)	7626	(30.4%)
	Present	10325	(80.0%)	719	(44.2%)	3949	(64.4%)	1879	(87.6%)	605	(26.4%)	17477	(69.6%)
Cough (any duration)	No	5952	(46.1%)	1013	(62.3%)	3339	(54.4%)	793	(37.0%)	1881	(82.0%)	12978	(51.7%)
	Yes	6955	(53.9%)	612	(37.7%)	2794	(45.6%)	1352	(63.0%)	412	(18.0%)	12125	(48.3%)
Cough (≥2 weeks)	No	9574	(74.2%)	1377	(84.7%)	4897	(79.8%)	1454	(67.8%)	2250	(98.1%)	19552	(77.9%)
	Yes	3333	(25.8%)	248	(15.3%)	1236	(20.2%)	691	(32.2%)	43	(1.9%)	5551	(22.1%)
Chest X-ray*	Active TB	134	(1.0%)	91	(5.6%)	788	(12.9%)	33	(1.5%)	7	(0.3%)	1053	(4.2%)
	TB activity undetermined	1901	(14.7%)	273	(16.8%)	1582	(25.8%)	291	(13.6%)	64	(2.8%)	4111	(16.4%)
	Inactive TB	342	(2.7%)	8	(0.5%)	182	(3.0%)	41	(1.9%)	3	(0.1%)	576	(2.3%)
	No TB findings	10524	(81.6%)	1253	(77.1%)	3580	(58.4%)	1780	(83.0%)	2219	(96.8%)	19356	(77.1%)
Suspected TB^†^	Not suspected	10831	(83.9%)	1260	(77.5%)	3744	(61.0%)	1821	(84.9%)	2222	(96.9%)	19878	(79.2%)
	Suspected	2076	(16.1%)	365	(22.5%)	2389	(39.0%)	324	(15.1%)	71	(3.1%)	5225	(20.8%)
Sputum smear test*	Negative	1598	(93.9%)	94	(79.7%)	1917	(89.8%)	189	(91.3%)	39	(92.9%)	3837	(91.3%)
	Positive	104	(6.1%)	24	(20.3%)	218	(10.2%)	18	(8.7%)	3	(7.1%)	367	(8.7%)
Xpert*	Negative	1800	(86.8%)	311	(91.5%)	1999	(84.7%)	261	(80.6%)	63	(92.6%)	4434	(85.8%)
	Positive	274	(13.2%)	29	(8.5%)	360	(15.3%)	63	(19.4%)	5	(7.4%)	731	(14.2%)
Rifampicin^‡^	Susceptible	257	(93.8%)	26	(89.7%)	329	(91.4%)	55	(87.3%)	5	(100.0%)	672	(91.9%)
	Resistant	16	(5.8%)	3	(10.3%)	29	(8.1%)	7	(11.1%)	0	(0.0%)	55	(7.5%)
	Intermediate	1	(0.4%)	0	(0.0%)	2	(0.6%)	1	(1.6%)	0	(0.0%)	4	(0.5%)
Concordance of lab results^§^	Smear + Xpert +	104	(6.1%)	23	(19.8%)	212	(10.0%)	18	(8.7%)	3	(7.1%)	360	(8.6%)
	Smear − Xpert +	168	(9.9%)	1	(0.9%)	146	(6.9%)	38	(18.4%)	1	(2.4%)	354	(8.4%)
	Smear + Xpert −	0	(0.0%)	0	(0.0%)	5	(0.2%)	0	(0.0%)	0	(0.0%)	5	(0.1%)
	Smear − Xpert −	1430	(84.0%)	92	(79.3%)	1767	(83.0%)	151	(72.9%)	38	(90.5%)	3478	(82.9%)
Final outcome	All forms of TB	284	(2.2%)	34	(2.1%)	378	(6.2%)	63	(2.9%)	5	(0.2%)	764	(3.0%)
	- Bacteriologically-confirmed TB	274	(2.1%)	30	(1.8%)	366	(6.0%)	63	(2.9%)	5	(0.2%)	738	(2.9%)
	- Clinically-diagnosed TB	10	(0.1%)	4	(0.2%)	12	(0.2%)	0	(0.0%)	0	(0.0%)	26	(0.1%)
	NNS	45		48		16		34		459		33	

* Cases with unknown or missing information were excluded from the calculation.

^†^ Suspected TB was determined by clinical judgement based primarily on X-ray results and additionally on TB symptoms.

^‡^ Among cases with positive Xpert results.

^§^ Among cases with results of both sputum smear and Xpert tests.

NNS: Number needed to screen to detect one TB patient (all forms of pulmonary TB)

One or more TB symptoms (any TB symptom) was identified in 69.6% of all participants, with the highest prevalence found in indigenous population (87.6%), followed by the rural poor (80.0%), prisons (64.4%), the urban poor (44.2%), and high schools (26.4%). Cough more than two weeks was identified in 22.1% of all participants, with the highest prevalence reported in indigenous population (32.2%), followed by the rural poor (25.8%), prisons (20.2%), the urban poor (15.3%) and high schools (1.9%).

Prison inmates had a high proportion of abnormal CXR results with suggestive of “active TB” (12.9%) and “TB activity undetermined” (25.8%). A lower proportion of CXR abnormality was found in other populations with the far lowest proportion observed in high school. Accordingly, the highest proportion of people with suspected TB was found in prison (39.0%), followed by the urban poor (22.5%), the rural poor (16.1%), indigenous population (15.1%) and high school (3.1%).

### Outcomes of diagnostic tests (Table 2)

A total of 764 patients with all forms of pulmonary TB (3.0% [95%CI 2.8–3.3%]) were detected by the project. Of them, 738 (96.6%) had bacteriologically-confirmed TB, and 26 (3.4%) had clinically-diagnosed TB. The highest yield of TB with the lowest NNS was found in prison (6.2% [95%CI 5.6–6.8%], NNS: 16), followed by indigenous population (2.9% [95%CI 2.3–3.7%], NNS: 34), the rural poor (2.2% [95%CI 2.0–2.5%], NNS: 45), the urban poor (2.1% [95%CI 1.5–2.9%], NNS: 48), and high school (0.2% [95%CI 0.1–0.5%], NNS: 459).

A total of 55 patients (7.5% of Xpert positive patients) had RR-TB, with the highest resistance rate found in indigenous population (11.1%). Of 55 RR-TB patients, 23 patients (41.8%) had no history of previous TB treatment. The highest proportion of smear-negative Xpert-positive patients among patients who had results of both tests was found in indigenous population (18.4%), followed by the rural poor (9.9%) and prisons (6.9%). Five patients (0.2%) in prisons were smear-positive Xpert-negative patients. All of them had abnormal results of CXR (two were suggestive of “active TB” while three were “TB activity undetermined”) and were diagnosed with TB based on the physician’s clinical judgement.

The highest smear positivity rate (the proportion of individuals with a smear-positive result among those who have smear results) was observed in the urban poor (20.3%). In contrast, the highest Xpert positivity rate (the proportion of individuals with an Xpert positive result among those who have Xpert results) was found in indigenous population (19.4%).

### Factors associated with diagnosis (yield) of TB (Tables 3–6)

**Table 3:** Yield of all forms of pulmonary TB by patient’s characteristics (rural poor communities).

Characteristics		Total	Confirmed TB			Univariate			Multivariate		
			Case	%	(95% CI)	Crude OR	(95% CI)	p-value	Adjusted OR	(95% CI)	p-value
Overall		12907	284	2.2	(1.9–2.4)						
Sex	Female	8055	84	1	(0.8–1.3)	1.00					
	Male	4852	200	4.1	(3.5–4.5)	4.08	(3.17–5.30)	<0.001***	2.3	(1.66–3.19)	<0.001***
Age category	15–24	1541	31	2	(1.4–2.8)	1.00					
	25–34	1991	31	1.6	(1.1–2.2)	0.77	(0.47–1.28)	0.309	0.58	(0.33–1.04)	0.066
	35–44	2465	50	2	(1.5–2.6)	1.01	(0.65–1.60)	0.971	0.77	(0.47–1.31)	0.328
	45–54	2491	68	2.7	(2.1–3.4)	1.37	(0.90–2.13)	0.154	0.86	(0.53–1.43)	0.558
	55–64	2214	58	2.6	(2.0–3.3)	1.31	(0.85–2.06)	0.230	0.62	(0.38–1.06)	0.073
	≥65	2205	46	2.1	(1.5–2.7)	1.04	(0.66–1.66)	0.874	0.48	(0.28–0.82)	0.006**
History of TB exposure	No	10007	204	2	(1.7–2.3)	1.00					
	Yes	2853	80	2.8	(2.2–3.4)	1.39	(1.06–1.79)	0.015*	1.17	(0.86–1.58)	0.300
History of previous TB treatment	No	11624	228	2	(1.7–2.2)	1.00					
	Yes	1213	56	4.6	(3.4–5.7)	2.42	(1.78–3.24)	<0.001***	1.29	(0.90–1.82)	0.153
Smoking status	No	7595	108	1.4	(1.2–1.7)	1.00					
	Yes	3103	143	4.6	(3.8–5.2)	3.35	(2.60–4.32)	<0.001***	1.7	(1.25–2.32)	<0.001***
Any TB symptoms	None	2582	14	0.5	(0.3–0.9)	1.00					
	Present	10325	270	2.6	(2.3–2.9)	4.93	(2.99–8.85)	<0.001***	1.63	(0.92–3.11)	0.115
Cough (≥2 weeks)	No	9574	89	0.9	(0.8–1.1)	1.00					
	Yes	3333	195	5.9	(4.8–6.3)	6.62	(5.16–8.57)	<0.001***	3.94	(2.93–5.35)	<0.001***
Fever	No	11779	209	1.8	(1.5–2.0)	1.00					
	Yes	1128	75	6.6	(5.0–7.7)	3.94	(2.99–5.15)	<0.001***	1.72	(1.23–2.38)	0.001**
Night sweats	No	10725	198	1.8	(1.6–2.1)	1.00					
	Yes	2182	86	3.9	(3.1–4.7)	2.18	(1.68–2.81)	<0.001***	1.24	(0.91–1.67)	0.170
Weight loss	No	11249	177	1.6	(1.3–1.8)	1.00					
	Yes	1658	107	6.5	(5.0–7.3)	4.32	(3.37–5.51)	<0.001***	2.37	(1.77–3.16)	<0.001***

CI: Confidence Interval, OR: Odds Ratio

* Significant difference (0.01 ≤ p < 0.05)

** Significant difference (0.001 ≤ p < 0.01)

*** Significant difference (p < 0.001)

**Table 4:** Yield of all forms of pulmonary TB by patient’s characteristics (urban poor communities).

Characteristics		Total	Confirmed TB			Univariate			Multivariate		
			Case	%	(95% CI)	Crude OR	(95% CI)	p-value	Adjusted OR	(95% CI)	p-value
Overall		1625	34	2.1	(1.5–2.9)						
Sex	Female	905	11	1.2	(0.7–2.1)	1.00					
	Male	720	23	3.2	(2.1–4.6)	2.68	(1.33–5.75)	0.008**	2.08	(0.98–4.62)	0.062
Age category	15–24	346	6	1.7	(0.8–3.7)	1.00					
	25–34	298	5	1.7	(0.7–3.8)	0.97	(0.28–3.24)	0.956	0.96	(0.26–3.41)	0.950
	35–44	341	10	2.9	(1.6–5.2)	1.71	(0.63–5.08)	0.303	1.67	(0.58–5.26)	0.351
	45–54	282	5	1.8	(0.8–4.0)	1.02	(0.29–3.43)	0.970	0.83	(0.22–2.94)	0.769
	55–64	205	4	2	(0.8–4.8)	1.13	(0.29–3.99)	0.854	0.49	(0.12–1.88)	0.307
	≥65	153	4	2.6	(1.0–6.4)	1.52	(0.38–5.40)	0.520	0.91	(0.21–3.51)	0.890
History of TB exposure	No	1559	33	2.1	(1.5–2.9)	1.00					
	Yes	63	1	1.6	(0.3–8.3)	0.75	(0.04–3.55)	0.774			
History of previous TB treatment	No	1498	28	1.9	(1.3–2.6)	1.00					
	Yes	120	6	5	(2.2–10.0)	2.76	(1.02–6.38)	0.027*	1.69	(0.57–4.27)	0.300
Smoking status	No	770	13	1.7	(1.0–2.8)	1.00					
	Yes	431	12	2.8	(1.6–4.7)	1.67	(0.74–3.71)	0.207			
Any TB symptoms	None	906	8	0.9	(0.4–1.7)	1.00					
	Present	719	26	3.6	(2.4–5.1)	4.21	(1.98–10.01)	<0.001***	0.93	(0.27–2.91)	0.905
Cough (≥2 weeks)	No	1377	13	0.9	(0.6–1.6)	1.00					
	Yes	248	21	8.5	(5.2–11.6)	9.71	(4.85–20.16)	<0.001***	6.73	(2.59–20.93)	<0.001***
Fever	No	1572	29	1.8	(1.3–2.6)	1.00					
	Yes	53	5	9.4	(3.7–18.6)	5.54	(1.83–13.82)	<0.001***	2.34	(0.66–6.96)	0.150
Night sweats	No	1565	27	1.7	(1.2–2.5)	1.00					
	Yes	60	7	11.7	(5.2–20.0)	7.52	(2.91–17.20)	<0.001***	2.41	(0.79–6.62)	0.102
Weight loss	No	1520	24	1.6	(1.1–2.3)	1.00					
	Yes	105	10	9.5	(4.8–15.3)	6.56	(2.92–13.75)	<0.001***	2.28	(0.88–5.59)	0.079

CI: Confidence Interval, OR: Odds Ratio

* Significant difference (0.01 ≤ p < 0.05)

** Significant difference (0.001 ≤ p < 0.01)

*** Significant difference (p < 0.001)

**Table 5:** Yield of all forms of pulmonary TB by patient’s characteristics (prison inmates).

Characteristics		Total	Confirmed TB			Univariate			Multivariate		
			Case	%	(95% CI)	Crude OR	(95% CI)	p-value	Adjusted OR	(95% CI)	p-value
Overall		6133	378	6.2	(5.3–6.4)						
Sex	Female	66	2	3	(0.8–10.1)	1.00					
	Male	6067	376	6.2	(5.3–6.4)	2.11	(0.66–12.91)	0.298	0.63	(0.18–3.97)	0.536
Age category	15–24	439	7	1.6	(0.8–3.2)	1.00					
	25–34	1443	49	3.4	(2.5–4.3)	2.17	(1.04–5.28)	0.058	1.42	(0.67–3.50)	0.397
	35–44	1878	138	7.3	(5.8–8.0)	4.89	(2.45–11.62)	<0.001***	2.43	(1.19–5.86)	0.027*
	45–54	1535	108	7	(5.5–7.9)	4.67	(2.32–11.13)	<0.001***	1.94	(0.94–4.72)	0.102
	55–64	699	69	9.9	(7.2–11.2)	6.76	(3.30–16.30)	<0.001***	2.27	(1.07–5.60)	0.048*
	≥65	139	7	5	(2.3–9.6)	3.27	(1.10–9.72)	0.029*	1.31	(0.43–4.00)	0.629
History of TB exposure	No	4647	261	5.6	(4.7–6.0)	1.00					
	Yes	1472	117	7.9	(6.2–8.8)	1.45	(1.15–1.82)	0.001**	0.86	(0.67–1.11)	0.267
History of previous TB treatment	No	5189	289	5.6	(4.7–5.9)	1.00					
	Yes	931	89	9.6	(7.1–10.6)	1.79	(1.39–2.29)	<0.001***	0.77	(0.57–1.01)	0.065
Smoking status	No	852	54	6.3	(4.6–7.7)	1.00					
	Yes	3980	262	6.6	(5.5–6.9)	1.04	(0.78–1.42)	0.793			
Prison	Prison A	688	15	2.2	(1.3–3.5)	1.00					
	Prison B	1453	28	1.9	(1.3–2.7)	0.88	(0.47–1.70)	0.697	0.92	(0.49–1.80)	0.797
	Prison C	2640	261	9.9	(8.0–10.1)	4.92	(3.01–8.71)	<0.001***	4.05	(2.41–7.32)	<0.001***
	Prison D	965	54	5.6	(4.1–6.8)	2.66	(1.53–4.92)	<0.001***	2.76	(1.55–5.22)	0.001**
	Prison E	343	17	5	(3.0–7.4)	2.34	(1.15–4.80)	0.018*	3.46	(1.65–7.30)	<0.001***
	Prison F	44	3	6.8	(2.2–17.2)	3.28	(0.74–10.45)	0.069	5.12	(1.13–16.82)	0.014*
Any TB symptoms	None	2184	61	2.8	(2.1–3.5)	1.00					
	Present	3949	317	8	(6.7–8.3)	3.04	(2.32–4.05)	<0.001***	1.41	(1.01–1.99)	0.048*
Cough (≥2 weeks)	No	4897	201	4.1	(3.4–4.5)	1.00					
	Yes	1236	177	14.3	(10.9–14.3)	3.90	(3.16–4.83)	<0.001***	2.41	(1.87–3.10)	<0.001***
Fever	No	5556	316	5.7	(4.8–6.0)	1.00					
	Yes	577	62	10.7	(7.6–12.2)	2.00	(1.49–2.64)	<0.001***	1.48	(1.06–2.04)	0.018*
Night sweats	No	5330	297	5.6	(4.7–5.9)	1.00					
	Yes	803	81	10.1	(7.4–11.2)	1.90	(1.46–2.45)	<0.001***	1.19	(0.89–1.59)	0.242
Weight loss	No	4715	185	3.9	(3.3–4.3)	1.00					
	Yes	1418	193	13.6	(10.5–13.7)	3.86	(3.12–4.77)	<0.001***	2.11	(1.65–2.71)	<0.001***

CI: Confidence Interval, OR: Odds Ratio

* Significant difference (0.01 ≤ p < 0.05)

** Significant difference (0.001 ≤ p < 0.01)

*** Significant difference (p < 0.001)

**Table 6:** Yield of all forms of pulmonary TB by patient’s characteristics (indigenous population).

Characteristics		Total	Confirmed TB			Univariate			Multivariate		
			Case	%	(95% CI)	Crude OR	(95% CI)	p-value	Adjusted OR	(95% CI)	p-value
Overall		2145	63	2.9	(2.2–3.6)						
Sex	Female	1376	23	1.7	(1.1–2.5)	1.00					
	Male	769	40	5.2	(3.6–6.7)	3.23	(1.93–5.51)	<0.001***	2.87	(1.71–4.93)	<0.001***
Age category	15–24	270	8	3	(1.5–5.6)	1.00					
	25–34	376	6	1.6	(0.7–3.4)	0.53	(0.17–1.54)	0.246	0.52	(0.17–1.54)	0.240
	35–44	447	11	2.5	(1.4–4.2)	0.83	(0.33–2.16)	0.685	0.74	(0.29–1.96)	0.533
	45–54	410	14	3.4	(2.0–5.5)	1.16	(0.49–2.93)	0.745	0.91	(0.37–2.37)	0.841
	55–64	325	14	4.3	(2.5–6.8)	1.47	(0.62–3.74)	0.389	1.15	(0.47–2.95)	0.768
	≥65	317	10	3.2	(1.7–5.5)	1.07	(0.41–2.83)	0.893	0.75	(0.28–2.03)	0.561
History of TB exposure	No	1714	48	2.8	(2.1–3.6)	1.00					
	Yes	421	15	3.6	(2.1–5.6)	1.28	(0.69–2.26)	0.409			
History of previous TB treatment	No	1930	50	2.6	(1.9–3.3)	1.00					
	Yes	189	13	6.9	(3.8–10.7)	2.78	(1.42–5.06)	0.001**	2.35	(1.18–4.38)	0.010*
Smoking status	No	991	19	1.9	(1.2–2.9)	1.00					
	Yes	479	16	3.3	(2.0–5.2)	1.77	(0.89–3.47)	0.098			
Province	Palawan	1934	54	2.8	(2.1–3.5)	1.00					
	Puerto Princesa	211	9	4.3	(2.2–7.6)	1.55	(0.71–3.04)	0.232			
Any TB symptoms	None	266	4	1.5	(0.6–3.8)	1.00					
	Present	1879	59	3.1	(2.4–3.9)	2.12	(0.87–7.04)	0.148			
Cough (≥2 weeks)	No	1454	31	2.1	(1.5–3.0)	1.00					
	Yes	691	32	4.6	(3.1–6.2)	2.23	(1.35–3.70)	0.002**	1.72	(1.02–2.91)	0.041*
Fever	No	1725	43	2.5	(1.8–3.3)	1.00					
	Yes	420	20	4.8	(3.0–6.9)	1.96	(1.12–3.32)	0.015*	1.41	(0.77–2.49)	0.252
Night sweats	No	1483	37	2.5	(1.8–3.3)	1.00					
	Yes	662	26	3.9	(2.6–5.5)	1.60	(0.95–2.65)	0.072			
Weight loss	No	1747	41	2.3	(1.7–3.1)	1.00					
	Yes	398	22	5.5	(3.5–7.8)	2.43	(1.41–4.09)	<0.001***	1.87	(1.05–3.26)	0.030*

CI: Confidence Interval, OR: Odds Ratio

* Significant difference (0.01 ≤ p < 0.05)

** Significant difference (0.001 ≤ p < 0.01)

*** Significant difference (p < 0.001)

Factors significantly associated with diagnosis of TB varied across different populations. In the rural poor, a significantly higher yield was found in male (AOR 2.3, 95%CI: 1.66–3.19), smokers (AOR 1.7, 95%CI: 1.25–2.32), and those with cough more than two weeks (AOR 3.94, 95%CI: 2.93–5.35), with fever (AOR 1.72, 95%CI: 1.23–2.38), and with weight loss (AOR 2.37, 95%CI: 1.77–3.16). In the urban poor, a significantly higher yield was found only in those with cough more than two weeks (AOR 6.73, 95%CI: 2.59–20.93). In prison, a significantly higher yield was found in middle-to-higher age groups; 35–44 (AOR 2.43, 95%CI: 1.19–5.86) and 55–64 (AOR 2.27, 95%CI: 1.07–5.60), four prison facilities (AOR ranged from 2.76 to 5.12), those with any TB symptoms (AOR 1.41, 95%CI: 1.01–1.99), with cough more than two weeks (AOR 2.41, 95%CI: 1.87–3.10), with fever (AOR 1.48, 95%CI: 1.06–2.04), and with weight loss (AOR 2.11, 95%CI: 1.65–2.71). In indigenous population, a significantly higher yield was found in male (AOR 2.87, 95%CI: 1.71–4.93), those with history of previous TB treatment (AOR 2.35, 95%CI: 1.18–4.38), those with cough more than two weeks (AOR 1.72, 95%CI: 1.02–2.91), and with weight loss (AOR 1.87, 95%CI: 1.05–3.26).

Cough more than two weeks showed a significant association in all target populations after adjustment. History of TB exposure and night sweats were not significantly associated with a higher yield in any of the target populations. Middle-to-higher age groups showed a significant association only in prison. History of previous TB treatment showed a significant association only in indigenous population. Smoking status showed a significant association only in the rural poor. Any symptom showed a significant association only in prison.

### Composition of TB patients by smear and symptom status

**Fig 1:**
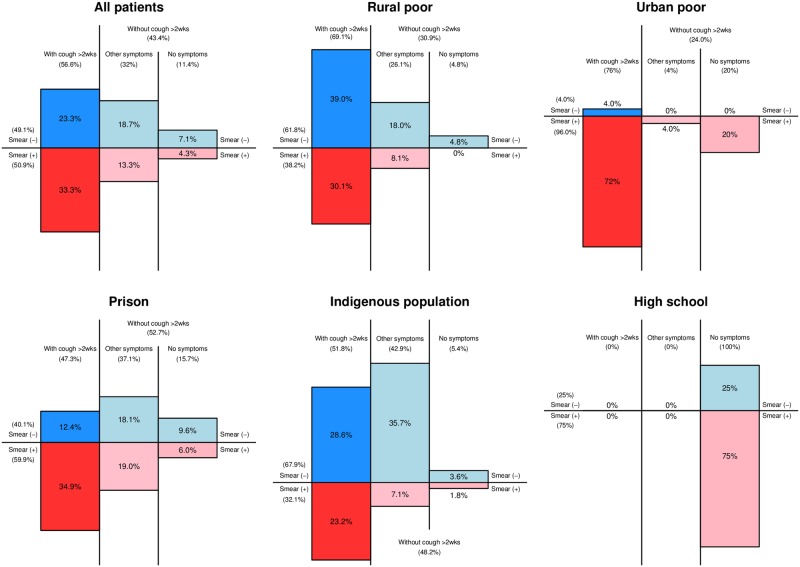
Composition of bacteriologically-confirmed TB patients by smear and symptom status, by target populations. Patients with unknown or missing sputum smear results were excluded from the calculation.

Fig 1 showed the composition of bacteriologically-confirmed TB patients by smear and symptom status for each target population. The proportion of smear-positive patients with cough more than two weeks was the highest in the urban poor (72.0%), followed by prison (34.9%), the rural poor (30.1%), indigenous population (23.2%), and high school (0%). The proportion of patients without cough more than two weeks was the highest in prison (52.7%), followed by indigenous population (48.2%), the rural poor (30.9%), and the urban poor (24.0%) (excluding 100% in high school due to a small number of patients). Among the four populations, the proportion of smear-negative (Xpert-positive) patients was low in the urban poor (4.0%) while it accounted for the substantial proportion in the rural poor (61.8%), prison (40.1%), and indigenous population (67.9%). The proportion of smear-negative patients without cough more than two weeks was the highest in indigenous population (39.3%), followed by prison (27.7%) and the rural poor (22.8%).

### Treatment outcomes (Table 7)

**Table 7:** Treatment outcomes of TB patients by target population.

Type of TB	Treatment outcomes	Rural poor		Urban poor		Prison inmates		Indigenous population		High school students		Total	
		N = 252	(%)	N = 23	(%)	N = 300	(%)	N = 57	(%)	N = 4	(%)	N = 636	(%)
Rifampicin-susceptible TB	Number of cohort	249		21		285		53		4		612	
	Treatment success	215	(86.3%)	20	(95.2%)	263	(92.3%)	46	(86.8%)	4	(100.0%)	548	(89.5%)
	- Cured	136	(54.6%)	6	(28.6%)	243	(85.3%)	27	(50.9%)	4	(100.0%)	416	(68.0%)
	- Completed	79	(31.7%)	14	(66.7%)	20	(7.0%)	19	(35.8%)	0	(0.0%)	132	(21.6%)
	Died	5	(2.0%)	1	(4.8%)	4	(1.4%)	1	(1.9%)	0	(0.0%)	11	(1.8%)
	Failure	3	(1.2%)	0	(0.0%)	3	(1.1%)	1	(1.9%)	0	(0.0%)	7	(1.1%)
	Loss to follow-up	16	(6.4%)	0	(0.0%)	0	(0.0%)	4	(7.5%)	0	(0.0%)	20	(3.3%)
	Transfer-out	10	(4.0%)	0	(0.0%)	15	(5.3%)	1	(1.9%)	0	(0.0%)	26	(4.2%)
Rifampicin-resistant TB	Number of cohort	3		2		15		4		0		24	
	Treatment success	1	(33.3%)	1	(50.0%)	14	(93.3%)	4	(100.0%)	0	(0.0%)	20	(83.3%)
	- Cured	0	(0.0%)	1	(50.0%)	2	(13.3%)	3	(75.0%)	0	(0.0%)	6	(25.0%)
	- Completed	1	(33.3%)	0	(0.0%)	12	(80.0%)	1	(25.0%)	0	(0.0%)	14	(58.3%)
	Died	0	(0.0%)	0	(0.0%)	1	(6.7%)	0	(0.0%)	0	(0.0%)	1	(4.2%)
	Failure	0	(0.0%)	0	(0.0%)	0	(0.0%)	0	(0.0%)	0	(0.0%)	0	(0.0%)
	Loss to follow-up	2	(66.7%)	1	(50.0%)	0	(0.0%)	0	(0.0%)	0	(0.0%)	3	(12.5%)
	Transfer-out	0	(0.0%)	0	(0.0%)	0	(0.0%)	0	(0.0%)	0	(0.0%)	0	(0.0%)

TSR was found to be high with 89.5% for patients with Rifampicin-susceptible (RS) TB and 83.3% for patients with RR-TB. For RS-TB patients, a higher TSR was observed in high school (100%), the urban poor (95.2%) and prison (92.3%). In contrast, a relatively higher loss to follow-up rate was reported in indigenous population (7.5%) and the rural poor (6.4%).

## Discussion

Our analysis showed a wide range of demographic profiles and prevalence of symptoms and predictors of TB across five different target populations. As a result, we found a wide variation of yield, ranging from 0.2% (NNS: 495) in high school, 2.1% (NNS: 48) in the urban poor, 2.2% (NNS: 45) in the rural poor, 2.9% (NNS: 34) in indigenous population and to 6.2% (NNS: 16) in prison. We also identified some commonalities and differences between target populations in the predictor analysis, composition of the patients by smear and symptom status, as well as in treatment outcomes.

In this project, screening in prison was found to be most effective among all target populations given the lowest NNS observed. A systematic review on NNS in TB screening showed that the median NNS for prisoners was 43 (IQR 21–123) in medium and high incidence settings which was among the lowest across different risk groups [10]. In the Philippines, excessive overcrowding in prisons has been well recognized [20], and mass-screening for TB has been conducted in some prisons in other regions of the country too, with a yield ranging from 2.5% (NNS: 40) to 3.2% (NNS: 31) [21,22]. Given that our project also employed mass screening in prisons, the results confirmed the high prevalence of TB among prison inmates in the Palawan settings. It is noteworthy that the prison with the highest yield had an over five-fold higher rate than the prison with the lowest yield (9.9% [NNS: 10] vs 1.9% [NNS: 53]), suggesting a wide range of TB prevalence across different prisons.

The highest NNS in high school suggested ineffectiveness as compared to other target populations. In the Philippines, the prevalence of culture positive TB for those aged 10–29 was estimated to be 0.28% [23], which was almost equivalent to the yield reported in high school students aged 15–23 (0.2%). School-based mass screening usually involves subjects from a specific age group, therefore an age-group specific prevalence rate from the national survey could represent expected yield in such screening activities. Although the prevalence in those with younger age varies depending on epidemiological profiles of countries, it is generally lower than that of general population in many countries [4,24–26] including the Philippines [23]. They are often categorized as a low risk group in which systematic screening for active TB is not recommended [9]. Some studies indeed showed a low yield in their TB screening among adolescents in high burden settings [27–29], being consistent with our results. However diagnosing and treating a younger patient would achieve more life-years saved than treating an older patient, and in some settings, may contribute to addressing health inequity and filling programmatic gaps. A study in Kenya demonstrated a high TB prevalence and low patient diagnostic rate in adolescents [30]. In such context with a strong evidence-base, screening adolescents including high school students may be justifiable. In the Palawan setting where TB is obviously less concentrated in high school students, a priority for ACF should be given to other populations with a higher risk of TB.

According to WHO, systematic screening for active TB may be considered for geographically defined subpopulations with extremely high levels of undetected TB (1% prevalence or higher), other subpopulations that have very poor access to health care, and other vulnerable or marginalized groups [8]. Our ACF among the urban poor, the rural poor and indigenous population is likely to fall in these categories, given the high yield of TB with more than 2%. In other countries with a high burden of TB, systematic screening also achieved a relatively high yield in the urban poor and slums [11,31–35], in the rural poor [13,36] and in indigenous population [37], being consistent with our results. These studies may not be directly comparable due to considerable differences in the definition of screening (denominator of the yield calculation), the disease prevalence in target population, and screening approaches including diagnostic tools and algorithms. In the Palawan setting, the high yield of TB among these populations could be explained by several factors. First, underlying TB prevalence was likely to be high due to poor access to health services and low socio-economic status. In particular, indigenous populations are likely to have the most limited health access as they usually reside in mountain areas [38], which could have led the highest yield among the three. Second, the robust diagnostic algorithm using a rapid sensitive test with a combination of digital CXR could have greatly increased the diagnostic accuracy. In the systematic review on NNS, inclusion of CXR in the screening tools was associated with low NNS [10], which supported our results. Furthermore, people who have TB symptoms could be more likely to visit the mobile unit for screening since the participation was based on a voluntary visit (except prison and high school screening where mass screening was conducted). This might have increased the yield by facilitating the spontaneous selection of people with a higher risk of TB. Indeed, the prevalence of TB symptomatics among them was higher than the national average of 13.3% reported in the 2007 national prevalence survey [23].

Our ACF approach was found to be effective in the four populations in terms of yield. However there are many other considerations in order to rationalize the project and further inform the continuation or scale-up of the ACF activities. First, cost-effectiveness and cost-utility of the project should be investigated and compared between target populations. Indicators such as cost per case detected and incremental cost-effectiveness ratio will provide important financial implications [39,40]. These cost-effectiveness indicators can also represent the efficiency of the screening including the average number of people screened per day and travel time and distance needed for screening. Second, the additionality of the ACF to case detection could be explored to evaluate the contribution of the project. Not all patients detected by the project can be regarded as true additional cases as some could have been detected by the routine PCF even if there was no project intervention [41]. An analysis of case notification trend in intervention areas before and during intervention periods as well as its comparison with control areas enables to estimate the number of true additional cases and provides a clearer picture of the contribution of ACF to increased case detection [13,42].

Our analysis of the composition of bacteriologically-confirmed TB by smear and symptom status enabled to quantify the contribution of CXR, Xpert and its combination to overall case detection. First, the patients without cough more than two weeks accounted for a substantial proportion, ranging from 24.0% to 52.7% (except high school students). If they underwent a routine health-seeking pathway, they would not have been presumed to have TB since the national guidelines use cough more than two weeks as criteria [17]. The use of CXR in initial screening helped identify many TB patients with mild or no symptoms. Given the nature of ACF where many asymptomatic individuals may be subject to screening [16], CXR plays a critically important role in ACF with its high sensitivity [43]. Positioning CXR in initial screening can be costly [44], however the use of digital X-ray machine, in place of conventional film X-ray, can significantly reduce running costs and simplify logistics [45], making it a feasible and cost-effective option even in a large scale ACF in resource-constrained programmes.

Second, the proportion of smear-negative (Xpert-positive) patients was high in three populations (61.8% in the rural poor, 40.1% in prison and 67.9% in indigenous population). They also would not have been detected in the routine diagnostic algorithm that employs sputum smear microscopy as a main tool for bacteriological confirmation [17]. Given that smear-negatives often indicate less severity of the disease [12,46,47], these patients might be detected at the early stage of their disease by using Xpert. In contrast, the high proportion of smear-positives was found in the urban poor, consistent with the results from the national prevalence survey [23]. This implies a considerable diagnostic delay in this population, which needs further investigation and addressing.

Third, smear-negative patients without cough more than two weeks also accounted for a substantial proportion in three populations (39.3% in indigenous population, 27.7% in prison and 22.8% in the rural poor). These patients would not have been diagnosed if either CXR or Xpert was not available in the project, thereby representing a shared contribution of CXR and Xpert. The contribution of CXR can be maximized when it is used in combination with Xpert, and vice versa. In our project, indigenous population appears to be most benefited from increased diagnostic sensitivity due to the combined use of CXR and Xpert. Less-severe and less-symptomatic TB patients may be most prevalent in this population.

In this project, the use of Xpert facilitates bacteriological confirmation among smear-negatives, which also could have led to the low proportion of clinically-diagnosed TB patients (3.4%). The national average in the proportion of clinically-diagnosed TB among all pulmonary TB patients was high with 64% in 2015 [1]. This may indicate the high detection of less or non-infectious cases but also reflect the issue of low sensitivity of sputum smear microscopy that is mainly used for bacteriological confirmation in the routine setting. Our results suggested that continued efforts to increase the availability of Xpert would make clinicians more convinced to rule out TB and reduce possible unnecessary TB treatment.

This study has several limitations. First, 5–10% of the participant records in the study period were missing, which could have influenced the study results. Second, some participants from indigenous population were selected from the defined areas of the rural poor communities. Hence their demographic and clinical characteristics could have showed some similarities with those of the rural poor. Third, five smear-positive Xpert-negative cases that we considered bacteriologically-confirmed cases may be possible non-tuberculosis mycobacteria, which could have slightly overestimated the yield in prison. Fourth, we highlighted the contribution of Xpert by showing the composition of patient by smear status. However our project used LED-FM that has a higher sensitivity than conventional light microscopy [48], which could have underestimated the contribution of Xpert. Finally, our study did not aim to achieve national representation for each target population therefore the results may not be generalized to other areas.

The results from our predictor analysis help provide additional screening criteria for each target population in the study setting. Our results showed a tendency that symptoms criteria had generally stronger association than demographic and other known risk factors. In particular, a significantly higher yield was found in those with cough more than two weeks in all populations. This reaffirmed the importance of continued emphasis on cough more than weeks that should remain the priority symptom criteria regardless of the presence of other risk factors. However focusing on only this symptom could miss a large number of cases as explained above. To find more cases missed in routine PCF, non- or less-symptomatic individuals should be included in screening based on other factors that had stronger associations with higher yield in addition to their relative risk of TB inherent in their respective populations. In our study setting, fever and weight loss were found to be more useful symptom criteria than night sweat. Demographic factors also have the potential to serve as effective screening criteria. Examining diagnostic power of each symptom by stratifying patient socio-demographic characteristics will enable to identify possible population-specific predictors of TB, which helps develop locally-defined screening criteria that can be used not only in ACF but also in routine PCF.

In this study, RS-TB patients had a TSR of 89.5%. This was almost equivalent to the TSR reported at the national level (92% for new and relapse cases in 2015) [1]. The TSR among RR-TB patients detected by the project was much higher than the national average of RR-/MDR-TB patients (83.3% vs 49%) [1], showing that ACF did not compromise treatment outcome. This finding is consistent with other studies [12,49] as well as a systematic review that concluded treatment outcome is similar between ACF and PCF [50]. However, the relatively lower TSR and higher loss to follow-up rate were found in indigenous population and the rural poor. In a study from South India, a higher loss to follow-up rate was particularly reported in smear-negative patients in ACF [16]. Given the higher proportion of smear-negative patients found in these two populations, they could have more patients with less severe disease, leading to higher loss to follow-up. The difference in accessibility to care across target populations could have also influenced treatment outcomes.

The United Nation’s Sustainable Development Goals (SDGs) aims to achieve Universal Health Coverage (UHC) which includes financial risk protection, access to quality essential health-care services and access to safe, effective, quality and affordable essential medicines and vaccines for all [51]. Well-designed ACF brings quality TB diagnostic service to high-risk and most vulnerable populations with no patient cost sharing, which could facilitate early case detection and help mitigate financial burden of the disease [52]. Such pro-poor case finding interventions intend to address health inequity, which is in line with the objectives of UHC and further supports achieving the SDGs. However, ACF, if poorly designed, may carry a risk of over-diagnosis [12] and false positive results due to less targeted screening involving people with low risk of TB [9], increasing unsuccessful treatment outcomes including initial loss to follow-up [16,50] and depriving resources for a poor yield. In this regard, careful assessment is essential to inform evidence-based decisions about target populations and diagnostic approaches that ensure the effectiveness of screening.

## Conclusions

In the Philippines, the ACF project that targeted vulnerable populations using mobile unit yielded a substantial number of TB patients and achieved successful treatment outcomes. In particular, screening in prison, indigenous population, and urban and rural poor communities was found to be effective and they can be considered high-risk populations in the study setting. The combined use of CXR and Xpert largely contributed to increased case detection. Further research is needed to explore cost-effectiveness of the project and diagnostic power of different symptoms to accelerate evidence-based interventions as well as to inform future TB screening policies in the Philippines.

## Supporting information

S1 Dataset Screening data from DetecTB project in Palawan, the Philippines. (XLSX)
